# Prioritized Criteria for Casualty Distribution following Trauma-related Mass Incidents; a Modified Delphi Study

**Published:** 2020-04-07

**Authors:** Mohammad Reza Khajehaminian, Ali Ardalan, Sayed Mohsen Hosseini Boroujeni, Amir Nejati, Omid Mahdi Ebadati, Mahdi Aghabagheri

**Affiliations:** 1 Department of Health in Emergencies and Disasters, School of Public Health, Shahid Sadoughi University of Medical Sciences and Health Services, Yazd, Iran.; 2 Department of Health in Emergencies and Disasters, School of Public Health, Tehran University of Medical Sciences, Tehran, Iran.; 3 Harvard Humanitarian Initiative, Harvard T.H. Chan School of Public Health, Cambridge, USA.; 4 School of Nursing (Broujen), Shahrekord University of Medical Sciences, Shahrekord, Iran.; 5 Department of Mathematics and Computer Sciences, Kharazmi University, Tehran, Iran.; 6 Meybod Nursing School, Shahid Sadoughi University of Medical Sciences, Yazd, Iran.

**Keywords:** Mass casualty incidents, wounds and injuries, decision making, supply and distribution

## Abstract

**Introduction:**

In the aftermath of mass casualty incidents (MCIs), many decisions need to be made in a fast and influential manner in a high pressure environment to distribute the limited resources among the numerous demands. This study was planned to rank the criteria influencing distribution of casualties following trauma-related MCI.

**Methods:**

This study utilized a modified Delphi methodology, concentrating on extracted criteria attained from preceding systematic literature reviews. The 114 extracted criteria were classified into eight sections including space, staff, equipment, system and structures, triage, treatment, transport, and uncategorized criteria and were imported into an online survey tool. In the first round, experts were asked to rank each criterion on a five-point Likert scale. The second round incorporated feedbacks from the first round, stating percent and median scores from the panel as a whole. Experts were then called upon to reassess their initial opinions regarding uncertain remarks from the first round, and once again prioritize the presented criteria.

**Results:**

Fifty-seven criteria were regarded as relevant to the following sections: space: 70% (7/10); staff: 44% (4/9); system / structure: 80% (4/5); equipment: 39.1% (9/23); treatment; 66.7% (6/9); triage: 73.7% (14/19); transport: 38.7% (12/31) and other sections: 12.5% (1/8). The ﬁrst round achieved nearly 98% (n=48) response rate. Of the 114 criteria given to the experts, 68 (almost 60%) were approved. The highest percentage of approval belonged to the system and structures sections (4/5=80%). The response rate for the second round was about 86% (n=42). A consensus could be reached about nearly 84% (57) of the 68 criteria presented to experts.

**Conclusion:**

“Casualty Level of Triage on the Scene” and “Number of Available Ambulances” were the two criteria that obtained the highest level of consensus. On the other hand, “gender of casualty”, “Number of Non-Medical staff in each Hospital” and “Desire to transport family members together” got lowest level of consensus. This sorted list could be used as a catalogue for developing a decision support system or tool for distribution of victims following mass casualty incidents.

## Introduction:

Victims of Mass Casualty Incidents (MCIs) ought to be distributed among the accessible hospitals so that no single hospital is excessively overloaded and at the same time casualty needs are met in accordance with hospitals abilities (1). Henceforth, in the aftermath of MCIs, many decisions need to be made in a fast and influential manner in a high pressure environment to distribute the limited resources among the numerous demands (2, 3). This process is comprised of multiple functions including triage, treatment and transport, which necessitate making numerous and complex decisions (4) and also allocating resources (5). 

Obviously, it is best to make any decision on the distribution of victims accurately and purposefully (6, 7). In this regard, efforts have been made to prepare a decision support system to assist prehospital and hospital emergency care managers and facilitate the distribution of casualties among available health care facilities (8-13). 

Although few studies have been conducted to identify influential criteria in distribution of casualties following MCIs (6, 7, 14), there is still a lack of prioritized criteria to guide decision makers in effective distribution of casualties. This study was planned to prioritize the criteria affecting the distribution of casualties following trauma-related MCIs.

## Methods:

***Study Design and setting***

This study utilized a modified Delphi methodology, concentrating on criteria extracted from a preceding systematic review (7). The major motives to apply this methodology were extensive use of this technique in health research, geographic spreading of experts and precluding the effects of noticeable view on the experts’ ideas (15). The study started in January and was completed in June 2018. 

***Participants***

The number of experts in Delphi panel can be 3 to 80 (16, 17); however, there is no universally agreed number of experts (18). In this study, the authors recognized 62 Iranian experts in the field of MCI management and they were asked to participate in the study. As some authors suggested (19, 20), a summary of research aims and probable Delphi rounds (2 rounds) and estimated time of assurance were verbally (face to face or via telephone) elucidated to the identified experts. Forty-nine experts consented to participate in this study and 48 of them completed first round. Six experts didn’t complete the second round. 

To avoid selection bias, the following criteria were used for choosing participants: (a) being affiliated in faculties, organization or institution engaged in incidents or disaster management (academic / researcher, and administrators of hospitals, prehospital emergency services or Red Crescent), (b) possessing at least 5 years of experience in disaster and incident management, (c) possessing clinical experience in trauma-related mass casualty incidents. These experts were chosen from emergency medicine specialists, emergency medical technicians, hospital physicians, nurses and midwifes. All experts had experience in MCI management, either in pre-hospital settings or within the hospital. It is highly suggested to select experts from various proficiencies and a wide geographic area (21). The experts’ characteristics are shown in table 1.

***Data Gathering***

In each round, research aims were presented clearly and experts were asked to prioritize the criteria presented in the questionnaire using a five-point Likert scale. A pilot study was done engaging two teachers in disaster medicine, and the reviewers made some minor modifications to the questionnaire statements before starting each round. Distribution of questionnaire and data gathering were conducted utilizing a web-based survey tool. The findings of the first round were presented to the experts as percentage and median of agreement rate on each statement in the first round. Three reminders were sent to those who had not responded. Since the findings of a preceding systematic literature review were utilized in this probe, two Delphi rounds were expected. There are no strict suggestions in the literature regarding the number of Delphi rounds, and the number of rounds is often predefined (21-23).

There is no clear suggestion for level of agreement in Delphi literature, but 75 % has been recommended as the minimum in some documents (21). Considering the large number of presented criteria, the classic inclusive approach of Delphi was not followed. Criteria that did not reach the minimum level of agreement were eliminated and the consensus criteria were included in the next step (exclusive approach).

Data gathering and processing were carried out at the end of each round. After the first round, the responses were merged; the respondents’ critical comments and effective feedback were assessed; and when appropriate, they were incorporated. We defined correctness as being related, practical and original. To answer any questions, the contact number of one of the researchers was included in the submitted questionnaire in each round.

***The Delphi procedures***

**Round 1**

The content of the first round questionnaire was based on the findings from a systematic literature review (7). All extracted criteria were listed as 114 statements classified into eight sections. The statements were imported to an online survey tool. The first section was relevant to experts’ characteristics, and the 8 following sections were (1) space, (2) staff, (3) equipment, (4) system and structures, (5) treatment, (6) triage, (7) transport, and (8) uncategorized criteria influencing casualties’ distribution following trauma-related MCI. In round 1 experts were asked to prioritize each criteria on a five-point Likert scale (Very high priority, High priority, Neutral, Low priority, Very low priority). There was a space for each section that the experts were encouraged to suggest additional criteria that they believed were missing.

For analysis, the five-point Likert scale was adapted to a three- point Likert scale with “1– 2” representing low priority, “3” representing neutral and “4-5” representing high priority, as recommended in other probes (19, 23-25). At least 75% of experts had to rank a criterion in the very high or high rank (score 4 or 5) for it to be chosen as a consensuses criterion.

Data were analyzed utilizing MS Excel to measure central tendency and dispersion indices. After analyzing the first round, minor modifications were applied to some statements according to participant comments in order to improve lucidity. Additionally, statements that were thought to be not in line with the objective of the study or were deemed a replication or were combinatory criteria were eliminated. In the subsequent round, based on feedbacks from the preceding round changes were incorporated. Before sending the questionnaires’ link to experts in any round, the reviewers judiciously reviewed its content and revised them where required.

**Round 2**

The questionnaire distributed in round 2 included all statements that experts had agreed upon. Round 2 incorporated feedbacks from Round 1 stated as percent and median scores from the panel as a whole. Experts were called upon to reassess their initial opinions on the statements from round 1, and once again prioritize presented criteria.

***Ethical consideration***

Experts were guaranteed that their responses to the questionnaire would be kept absolutely confidential, but participants were aware of the presence of other experts. This situation can be named as “quasi-anonymity” (20) and it is an inducement to participate in the Delphi study and can improve the response rate (21, 26). This research has been ethically confirmed by Institute Review Board of Tehran University of Medical Sciences with the registration Number IR.TUMS.SPH.REC.1395.509.

## Results:

Characteristics of study participants are described in table 1. The ﬁrst and second round achieved 98% and 86% response rate, respectively. Out of the 114 presented criteria, 57 criteria were accepted in the following sections: space: 70% (7/10); staff: 44% (4/9); system / structure: 80% (4/5); equipment: 39.1% (9/23); treatment; 66.7% (6/9); triage: 73.7% (14/19); transport: 38.7% (12/31) and other sections: 12.5% (1/8), (Table 2).

**Round 1**

To improve the reliability of the study, the investigators gathered the raw data and then raw data were analyzed by an analyzer blinded to the mentioned process. The criteria affecting casualty distribution in trauma-related MCI, based on a previous study (7) are listed in table 3. To clarify the findings, a code is given to each criterion. 

From the 62 identified experts, 49 agreed to participate in the study. In the first round, 114 criteria were given to the experts, 68 (almost 60%) of which were approved. The highest percentage of agreement belonged to system and structures sections (4/5=80%) and the agreement rate in other sections were as follows: space (7/10=70%), staff (4/9=44%), equipment (13/23=57%), treatment (6/9=67%), triage (14/19=74%), transport (16/31=52%), uncategorized criteria (4/8=50%).

Based on experts’ feedbacks, the following modifications were applied and then the flawed items were removed from the list (Code 23 due to being too general), (Codes 26, 29, 31 were replications of 27), (Code 68 was replication of 39 and 43), (Code 85 was replication of 86) and (codes 104, 113, 114 were represented by some other criteria). Overall, 37 of the 114 criteria could not achieve the consensus and were eliminated. No extra criterion was suggested by experts. As explained earlier, considering to the aim of study, the classic approach of Delphi study was not followed and accepted criteria (n=68) were included in the questionnaire for the second round. 

**Round 2**

From the 68 criteria presented to experts, about 84% (57) could obtain consensus. All presented criteria in space (n=7), staff (n=4), system and structure (n=4), treatment (n=6), and triage (n=14) could obtain consensus. The consensus rates in other sections were as follows: equipment (9/13≊69%), transport (12/16=75%), uncategorized criteria (1/4=25%). After completion of the second round, it was decided that a consensus had been obtained and further rounds were not required.

## Discussion:

This study prioritized the criteria affecting decision making for distribution following mass casualty incidents and found 57 high ranked criteria in this regard. The response rate reached 98% in the first round and 86% in the second round. 

Although decrease in the number of participants in the second round may have many justifications, it could be ascribed to the large number -114 criteria- of assessed statements. If a certain portion of participants refuse to continue a Delphi study, findings will be disturbed (15). Nonetheless, in this study, the number of participants had not greatly altered between the 2 rounds and therefore, findings were reliable (table 1).

Finally, accepted criteria in round 2 (table 4) were all sorted in accordance with the level of agreement. The level of consensus for each accepted criteria may suggest how a certain criterion is affecting decision-making. “Casualty Level of Triage on the Scene” and “Number of Available Ambulances” were the two criteria that obtained the maximum level of consensus (100%). On the other hand, “gender of casualty” (4.2%), “Number of Non-Medical staff in each Hospital” (20.8%) and “Desire to transport family members together” (20.8%) had the lowest level of consensus.

**Table 1:** Baseline characteristics of experts who completed the Delphi rounds

Variables	Number of participants	
	Round1 (n=48)	Round2 (n=42)
Gender		
**Female**	6 (12.5)	6 (14.3)
**Male**	42 (87.5)	36 (85.7)
Level of education		
**Bachelor’s Degree**	11 (22.9)	7 (16.7)
**Master’s Degree or MD**	29 (60.4)	27 (64.3)
**Ph.D. or Medical Specialist**	8 (16.7)	8 (19.0)
Field of study		
**Prehospital emergency care**	5 (10.4)	4 (9.5)
**Nurse**	23 (47.9)	19 (47.6)
**Midwife**	1 (2.1)	1 (2.4)
**Physician**	19 (39.6)	17 (40.5)
Professional/employment		
**EOC officer**	8 (16.7)	5 (11.9)
**Prehospital administrator**	12 (25.0)	11 (26.2)
**Hospital administrator**	7 (14.6)	5 (16.7)
**Academic / Researcher**	12 (25)	12 (28.6)
**Emergency medicine specialist**	7 (14.6)	7 (16.7)
**Red Crescent Administrator**	2 (4.2)	2 (4.8)
Age (year)		
**Mean ± SD**	41.7 ± 6.3	40.8 ± 5.9
Length of experience (years)		
**Mean ± SD**	10.2 ± 5.2	9.7 ± 4.6
**5-10 **	25 (52.1)	23 (54.8)
**10-15 **	15 (31.3)	28.6)
**15-20 **	7 (14.6)	6 (14.3)
**>20 **	1 (2.1)	1 (2.4)

Data are presented as mean ± standard deviation (SD) or frequency (%);

EOC: Emergency Operation Center

**Table 2:** The status of criteria in each Delphi rounds according to each section

Criteria	Accepted Criteria		
	Round 1	Round 2	Total
**Space**	70% (7/10)	100% (7/7)	70% (7/10)
**Staff**	44% (4/9)	100% (4/4)	44% (4/9)
**System / Structure**	80% (4/5)	100% (4/4)	80% (4/5)
**Equipment**	57% (13/23)	69% (9/13)	31.9% (9/23)
**Treatment**	67% (6/9)	100% (6/6)	66.7% (6/9)
**Triage**	74% (14/19)	100% (14/14)	73.7% (14/19)
**Transport**	52% (16/31)	75% (12/16)	38.7% (12/31)
**Uncategorized**	50% (4/8)	25% (1/4)	12.5% (1/8)

**Table 3:** The status of all presented criteria in each round

Status	Round2			Round1			All Criteria (n = 114)	
	SD	Mean	LOC	SD3	Mean	LOC		
							**Space**	
**Accepted**	0.84	4.21	86	0.73	4.21	81.25	Number of Involved Hospitals (9, 10, 28-36)	1
**3&**	0.63	4.50	92	0.67	4.44	89.58	Number of Available Hospitals (13, 36-39)	2
**R1 Rejected**				0.93	3.21	35.42	Number of Eligible Alternative Health Care Facilities (40)	3
**Accepted**	0.72	4.02	76	0.75	4.17	83.33	Hospital Bed Occupancy Rate (10)	4
**R1 Rejected**				0.79	3.79	68.75	Patient Presentation Rate of each Hospital (32, 41, 42)	5
**Accepted**	0.62	4.17	88	0.83	4.13	83.33	HACSC (Hospital Acute Care Surge Capacity) (34, 43)	6
**Accepted**	0.76	4.05	79	0.71	4.04	85.42	HACSC6 (Hospital Acute Care Surge Capacity in 6 hour) (34)	7
**Accepted**	0.73	4.05	81	0.77	3.90	81.25	HBSC (Hospital bed surge capacity) (34)	8
**Accepted**	0.75	4.07	76	0.89	3.85	81.25	HACST (Hospital Acute Care Surge Threshold) (34)	9
**R1 Rejected**				0.90	3.83	72.92	Capacity Factor (44)	10
Staff								
**Accepted**	0.80	4.50	90	0.87	4.31	81.25	Number of Nurses in each Hospital (31-33, 41, 45)	11
**Accepted**	0.91	4.05	76	0.91	4.15	77.08	Number of Physicians in each Hospital (31-33, 36, 39, 41, 46)	12
**R1 Rejected**				0.87	3.83	68.75	Number of Critical Care Nurses in each Hospital (8)	13
**R1 Rejected**				0.94	3.83	62.5	Number of Critical Care Physicians in each Hospital (8)	14
**Accepted**	0.92	4.29	86	1.08	4.21	79.17	Number of on-scene Emergency Medical Technicians (36, 37, 45)	15
**Accepted**	0.85	4.38	86	0.92	4.31	87.5	Number of Surgeons in each Hospital (8, 33, 36, 41)	16
**R1 Rejected**				1.14	3.21	43.75	Number of on-scene Physicians (36)	17
**R1 Rejected**				1.19	3.10	31.25	Number of on-scene Specialist Physicians (47)	18
**R1 Rejected**				0.98	2.96	20.83	Number of Non-Medical staffs in each Hospital (41)	19
							**System / Structure**	
**Accepted**	0.97	4.29	81	0.67	4.38	93.75	Hospital Level of Trauma (9, 10, 28, 31, 32, 35-39, 42, 48, 49)	20
**Accepted**	0.86	4.29	83	0.83	4.35	85.42	Activation of Hospital Disaster Plan (28, 29, 32, 34, 41, 47)	21
**Accepted**	0.87	4.02	81	0.71	4.04	77.08	Specialized Department in each Hospital (9, 28, 29, 32, 33, 37, 45, 49)	22
**R1 Omitted**				0.76	4.29	89.58	Hospital Capability (9, 29-31, 37, 39, 40, 45, 47)	23
**Accepted**	0.78	4.14	81	0.83	4.19	85.42	Number of Rapid Response/Trauma Teams in each hospital (32)	24
							**Equipment**	
**R2 Rejected**	0.82	3.76	67	0.73	4.21	85.42	Total Number of Beds in each Hospital (8, 28, 36, 37, 39, 46)	25
**R1 Omitted**				0.74	4.23	85.42	Real Time Hospital Bed Capacity (9, 10, 13, 29-32, 37, 39-41, 46, 49)	26
**Accepted**	0.84	4.21	83	0.70	4.40	87.5	Real Time Hospital Bed Capacity for each Level of Triage (31, 49)	27
**R2 Rejected**	0.83	3.71	52	0.53	3.92	81.25	Total No. of General Ward Beds in each Hospital (33)	28
**R1 Omitted**				0.79	4.08	81.25	Number of Available General Ward Beds in each Hospital (33)	29
**Accepted**	0.63	4.50	93	0.69	4.06	83.33	Total Number of ICU Beds in each Hospital (8, 28, 32, 36, 42, 45)	30
**R1 Omitted**				0.77	4.33	89.58	Number of Available ICU Beds in each Hospital (33)	31
**Accepted**	0.59	4.50	95	0.70	4.40	87.5	Number of Operating Rooms in each Hospital (28, 31, 32, 36, 37, 42, 50)	32
**Accepted**	0.71	4.50	88	0.76	4.46	91.67	Number of Available Operating Rooms in each Hospital (8, 45)	33
**Accepted**	0.81	3.98	79	0.69	4.06	87.5	Number of Ventilators in each Hospital (33, 42)	34
**Accepted**	0.94	4.05	76	0.82	4.29	89.58	Number of Available Ventilators in each Hospital (41)	35
**R1 Rejected**				0.85	3.75	60.42	Number of Recovery Beds in each Hospital (28)	36
**R1 Rejected**				0.80	3.67	54.17	Number of Bedside Cardiac Monitors in each Hospital (28, 45)	37
**R1 Rejected**				0.90	3.83	62.5	Number Available Bedside Cardiac Monitors in each Hospital (41)	38
**R2 Rejected**	0.98	3.90	69	0.80	4.17	79.17	Number of X-Ray Machines in each Hospital (31, 48, 50)	39
**R2 Rejected**	0.98	3.79	62	0.87	4.04	77.08	CT-Scan Availability in each Hospital (8, 45)	40
**R1 Rejected**				1.08	2.92	22.92	MRI Availability in each Hospital (31)	41
**Accepted**	0.50	4.74	98	0.67	4.63	93.75	Number of Emergency Department Beds in each Hospital (31, 34, 36)	42
**Accepted**	0.88	4.05	79	0.93	4.21	83.33	Number of Resuscitation Beds in Emergency Department of each Hospital (45, 48)	43
**Accepted**	0.93	4.10	79	0.88	4.40	87.5	Number of Trauma Rooms in Emergency Department of each Hospital (32, 45)	44
**R1 Rejected**				0.98	4.00	70.83	Amount of Pharmaceutical Supply in each Hospital (33)	45
**R1 Rejected**				0.92	3.65	60.42	Amounts Consumables Supply in each Hospital (33, 45)	46
**R1 Rejected**				1.06	3.56	56.25	Amount of Pre-hospital Medical Supply (33, 45)	47
							**Treatment**	
**R1 Rejected**				1.12	3.67	60.42	On-scene Treatment Time (13, 31)	48
**R1 Rejected**				0.92	3.77	62.5	Hospital Treatment Time (9, 31, 34, 50, 51)	49
**Accepted**	0.61	4.33	93	0.70	4.40	87.5	Casualty’s Need for Surgical Treatment in Hospital (35, 36)	50
**Accepted**	0.67	4.43	90	0.74	4.23	85.42	Casualty’s Need for On-scene Stabilizing Treatment (29, 36)	51
**Accepted**	0.66	4.40	90	0.78	4.35	85.42	Availability of On-scene Treatment (47)	52
**Accepted**	0.73	4.26	88	0.85	4.35	87.5	On-Scene Treatment Impact (47)	53
**R1 Rejected**				0.88	3.88	72.92	Expected Number of Lifesaving Surgeries in each Hospital (49)	54
**Accepted**	0.70	4.17	83	0.79	4.21	81.25	Number of Casualties in Needed of Ventilator (35)	55
**Accepted**	0.65	4.33	90	0.68	4.29	87.5	Number of Casualties in Needed of ICU Care Units (35)	56
Triage								
**Accepted**	0.33	4.88	100	0.58	4.71	93.75	Casualty’s Level of Triage on the Scene (9-13, 28, 30-33, 35-42, 47, 48, 50, 51)	57
**Accepted**	0.71	4.48	88	0.61	4.54	93.75	Casualty’s Level of Triage at Hospital (10, 32, 36, 48)	58
**R1 Rejected**				0.74	3.77	66.67	Over Triage Rate of Casualties (10, 36, 48)	59
**R1 Rejected**				0.85	3.77	62.5	Under Triage Rate of Casualties (10, 36, 48)	60
**Accepted**	0.67	4.19	86	0.69	4.25	85.42	Trauma Score of Casualty (8, 11, 12, 31-33, 35, 36, 38, 39, 42, 45, 46, 50, 51)	61
**Accepted**	0.68	4.31	88	0.67	4.44	89.58	Number of Casualties in each Triage Level (11, 12, 31, 33, 34, 38-41, 48-51)	62
**Accepted**	0.63	4.19	88	0.73	4.29	87.5	Vital Signs (BP, RR, PR) of Casualty (13, 33, 41, 47)	63
**Accepted**	0.90	3.98	79	0.79	4.29	87.5	Survival Probability of Casualty (31, 33, 39, 50, 51)	64
**Accepted**	0.75	3.98	81	0.81	4.19	83.33	Casualty’s Deterioration Rate (31, 33)	65
**Accepted**	0.59	4.45	95	0.68	4.46	89.58	Total Number of Casualties (11-13, 28, 31, 33, 37, 38, 41, 45, 48-51)	66
**R1 Rejected**				0.91	3.44	50	Pulse Oximetry of Casualty (13, 33, 41)	67
**R1 Omitted**				0.80	4.02	85.42	Physical Examination Findings of Casualty (13, 36, 42, 49)	68
**Accepted**	0.66	4.17	86	0.77	4.23	83.33	GCS of Casualty (33, 41, 47)	69
**Accepted**	0.79	4.05	81	0.66	4.33	89.58	Casualty's Type of Injuries (8, 12, 13, 32, 33, 35, 37, 38, 42, 47-49, 51)	70
**Accepted**	0.81	4.14	79	0.81	4.44	87.5	Pregnancy Status of Female Casualty (42, 47)	71
**Accepted**	0.66	4.26	88	0.80	4.33	87.5	Number of Child Casualties (47)	72
**Accepted**	0.83	4.12	76	0.88	4.06	77.08	Number of severe/moderate patients admitted to surgical departments in the last 24 hours in each hospital (8, 34)	73
**Accepted**	0.86	4.52	93	0.93	4.42	89.58	Possibility of Casualty’s Contamination (13, 28, 41)	74
**R1 Rejected**				1.04	3.52	50	Casualty's Age (13, 33, 47)	75
							**Transportation**	
**Accepted**	0.67	4.19	86	0.96	4.17	81.25	Incident Location (8-13, 28, 29, 32, 36, 38, 39, 42, 48, 49)	76
**Accepted**	0.73	4.26	88	0.71	4.23	83.33	Hospital Location (9, 10, 29, 32, 38, 39)	77
**Accepted**	0.67	4.43	90	0.73	4.44	89.58	Distance from MCI Location to each Hospital (31, 32, 36-39, 41, 45, 46, 48, 49)	78
**R2 Rejected**	0.94	4.19	74	0.75	4.33	83.33	Medical Center in Close Proximity of Incident (9, 48, 49)	79
**Accepted**	0.75	4.21	86	0.86	4.27	85.42	Location of EMS Stations (30)	80
**Accepted**	0.81	4.29	83	0.64	4.42	95.83	Available Means of Transportation (13, 37, 41)	81
**Accepted**	0.50	4.60	100	0.44	4.73	100	Number of Available Ambulances (30-33, 35-37, 39, 41, 42, 45, 47-49)	82
**R1 Rejected**				0.86	3.88	64.58	Type of Ambulance (33, 36, 37, 39, 42, 46-48)	83
**R1 Rejected**				0.72	3.94	70.83	Number of patients that can be transported by Ambulance simultaneously (39, 41)	84
**R1 Omitted**				0.71	4.04	77.08	Estimated Driving Time from Scene to each Hospital (8-10, 29, 32, 36, 39, 47)	85
**Accepted**	0.88	4.10	76	0.75	4.13	81.25	Round Trip Time for Ambulances (31)	86
**Accepted**	0.76	4.24	86	0.64	4.27	89.58	Number of Casualty Buses (32)	87
**R1 Rejected**				0.85	3.94	72.92	The Quality of Roads (48)	88
**Accepted**	0.76	4.17	79	0.70	4.38	87.5	Traffic Information (48)	89
**R2 Rejected**	1.16	3.93	67	0.94	4.10	79.17	Number of Available Helicopters (32, 35-37, 48, 49)	90
**R2 Rejected**	1.04	3.74	62	0.93	4.04	77.08	Maximum Capacity of each Helicopter (36)	91
**R2 Rejected**	1.07	3.98	69	0.93	4.04	77.08	Helicopter Landing Area near the Incident Location (28, 41, 49)	92
**R1 Rejected**				0.85	3.90	72.92	The distance from closest Helicopter Landing Area to the Scene (36)	93
**Accepted**	0.98	4.10	79	0.98	4.21	83.33	Helicopter Landing Place near Hospital (41)	94
**R1 Rejected**				0.92	3.77	62.5	Estimated time for each HEMS Mission/epoch (32, 36)	95
**R1 Rejected**				1.09	3.65	58.33	Possibility of fixed wing utilization in casualties’ evacuation (36)	96
**R1 Rejected**				1.10	3.56	54.17	Number of fixed wing aircrafts (36)	97
**R1 Rejected**				1.08	3.60	56.25	Maximum Capacity of each Fixed wing aircraft (36)	98
**R1 Rejected**				0.88	3.73	56.25	Number Casualties in need of secondary Transfer (35, 48)	99
**R1 Rejected**				0.93	3.73	68.75	Occurrence of the incident near the geographical border of disaster management (32)	100
**Accepted**	0.67	4.19	90	0.87	4.31	85.42	Injury to Hospital Interval (33, 35, 39, 44, 46)	101
**R1 Rejected**				0.89	3.54	50	The Last Time of Casualty delivery to the Determined Hospital (10)	102
**Accepted**	0.76	4.17	83	0.80	4.35	83.33	Injury to Patient Contact Interval (33, 44)	103
**R1 Omitted**				0.68	4.48	89.58	TF (Time Factor: the estimated proportion of critical and moderate patients with an IHI under the MTA) (44)	104
**R1 Rejected**				0.96	2.96	20.83	Desire to Transport family members together (8)	105
**R1 Rejected**				0.94	3.77	62.5	Number of Self Referencing Casualties to Hospital (28, 29, 32, 34, 35, 38, 40, 42, 44, 49)	106
							**Uncategorized**	
**R2 Rejected**	0.94	3.74	62	0.81	4.27	81.25	Mechanism of Injury (41)	107
**R2 Rejected**	0.94	3.95	74	0.78	4.25	83.33	Type of Incident (33, 41)	108
**R1 Rejected**				0.92	2.31	4.167	Gender of Casualty (13, 33, 47)	109
**Accepted**	0.71	4.29	90	0.64	4.40	91.67	Severity of Incident (Burden of Casualties) (41, 43)	110
**R1 Rejected**				0.84	3.71	58.33	Time of incident (11, 38, 40, 42)	111
**R2 Rejected**	0.91	3.95	71	0.81	4.08	75	Casualty’s Need for Extrication (29, 35)	112
**R1 Omitted**				0.72	4.33	93.75	TMC (Total Medical Capacity) (43)	113
**R1 Omitted**				0.66	4.17	89.58	R (Medical Rescue Capacity) (44)	114

LOC: Level of Consensus; SD = Standard Deviation.

**Table 4:** All accepted criteria in Delphi study prioritized based on level of consensus

SD	Mean	LOC	Criteria	Code	Section
**0.33**	4.88	100	Casualty’s Level of Triage on the Scene (9-13, 28, 30-33, 35-42, 47, 48, 50, 51)	57	Triage
**0.50**	4.60	100	Number of Available Ambulances (30-33, 35-37, 39, 41, 42, 45, 47-49)	82	Transport
**0.50**	4.74	98	Number of Emergency Department Beds in each Hospital (31, 34, 36)	42	Equipment
**0.59**	4.50	95	Number of Operating Rooms in each Hospital (28, 31, 32, 36, 37, 42, 50)	32	Equipment
**0.59**	4.45	95	Total Number of Casualties (11-13, 28, 31, 33, 37, 38, 41, 45, 48-51)	66	Triage
**0.63**	4.50	93	Total Number of ICU Beds in each Hospital (8, 28, 32, 36, 42, 45)	30	Equipment
**0.61**	4.33	93	Casualty’s Need for Surgical Treatment in Hospital (35, 36)	50	Treatment
**0.86**	4.52	93	Possibility of Casualty Contamination (13, 28, 41)	74	Triage
**0.63**	4.50	92	Number of Available Hospitals (13, 36-39)	2	Space
**0.80**	4.50	90	Number of Nurses in each Hospital (31-33, 41, 45)	11	Staff
**0.67**	4.43	90	Casualty’s Need for On-scene Stabilizing Treatment (29, 36)	51	Treatment
**0.66**	4.40	90	Availability of On-scene Treatment (47)	52	Treatment
**0.65**	4.33	90	Number of Casualties in need of ICU Care Units (35)	56	Treatment
**0.67**	4.43	90	Distance from MCI Location to each Hospital (31, 32, 36-39, 41, 45, 46, 48, 49)	78	Transport
**0.67**	4.19	90	Injury to Hospital Interval (33, 35, 39, 44, 46)	101	Transport
**0.71**	4.29	90	Severity of Incident (Burden of Casualties) (41, 43)	110	Uncategorized
**0.62**	4.17	88	HACSC (Hospital Acute Care Surge Capacity) (34, 43)	6	Space
**0.71**	4.50	88	Number of Available Operating Rooms in each Hospital (8, 45)	33	Equipment
**0.73**	4.26	88	On-Scene Treatment Impact (47)	53	Treatment
**0.71**	4.48	88	Casualty’s Level of Triage at Hospital (10, 32, 36, 48)	58	Triage
**0.68**	4.31	88	Number of Casualties in each Triage Level (11, 12, 31, 33, 34, 38-41, 48-51)	62	Triage
**0.63**	4.19	88	Vital Signs (BP, RR, PR) of Casualty (13, 33, 41, 47)	63	Triage
**0.66**	4.26	88	Number of Child Casualties (47)	72	Triage
**0.73**	4.26	88	Hospital Location (9, 10, 29, 32, 38, 39)	77	Transport
**0.84**	4.21	86	Number of Involved Hospitals (9, 10, 28-36)	1	Space
**0.92**	4.29	86	Number of on-scene Emergency Medical Technicians (36, 37, 45)	15	Staff
**0.85**	4.38	86	Number of Surgeons in each Hospital (8, 33, 36, 41)	16	Staff
**0.67**	4.19	86	Trauma Score of Casualty (8, 11, 12, 31-33, 35, 36, 38, 39, 42, 45, 46, 50, 51)	61	Triage
**0.66**	4.17	86	GCS of Casualty (33, 41, 47)	69	Triage
**0.67**	4.19	86	Incident Location (8-13, 28, 29, 32, 36, 38, 39, 42, 48, 49)	76	Transport
**0.75**	4.21	86	Location of EMS Stations (30)	80	Transport
**0.76**	4.24	86	Number of Casualty Buses (32)	87	Transport
**0.86**	4.29	83	Activation of Hospital Disaster Plan (28, 29, 32, 34, 41, 47)	21	System*
**0.84**	4.21	83	Real Time Hospital Bed Capacity for each Level of Triage (31, 49)	27	Equipment
**0.70**	4.17	83	Number of Casualties in need of Ventilator (35)	55	Treatment
**0.81**	4.29	83	Available Means of Transportation (13, 37, 41)	81	Transport
**0.76**	4.17	83	Injury to Patient Contact Interval (33, 44)	103	Transport
**0.73**	4.05	81	HBSC (Hospital bed surge capacity) (34)	8	Space
**0.97**	4.29	81	Hospital Level of Trauma (9, 10, 28, 31, 32, 35-39, 42, 48, 49)	20	System
**0.87**	4.02	81	Specialized Department in each Hospital (9, 28, 29, 32, 33, 37, 45, 49)	22	System
**0.78**	4.14	81	Number of Rapid Response/Trauma Teams in each hospital (32)	24	System
**0.75**	3.98	81	Casualty’s Deterioration Rate (31, 33)	65	Triage
**0.79**	4.05	81	Casualty's Type of Injuries (8, 12, 13, 32, 33, 35, 37, 38, 42, 47-49, 51)	70	Triage
**0.76**	4.05	79	HACSC6 (Hospital Acute Care Surge Capacity in 6 hour) (34)	7	Space
**0.81**	3.98	79	Number of Ventilators in each Hospital (33, 42)	34	Equipment
**0.88**	4.05	79	Number of Resuscitation Beds in Emergency Department of each Hospital (45, 48)	43	Equipment
**0.93**	4.10	79	Number of Trauma Rooms in Emergency Department of each Hospital (32, 45)	44	Equipment
**0.90**	3.98	79	Survival Probability of Casualty (31, 33, 39, 50, 51)	64	Triage
**0.81**	4.14	79	Pregnancy Status of Female Casualty (42, 47)	71	Triage
**0.76**	4.17	79	Traffic Information (48)	89	Transport
**0.98**	4.10	79	Helicopter Landing Place near Hospital (41)	94	Transport
**0.72**	4.02	76	Hospital Bed Occupancy Rate (10)	4	Space
**0.75**	4.07	76	HACST (Hospital Acute Care Surge Threshold) (34)	9	Space
**0.91**	4.05	76	Number of Physicians in each Hospital (31-33, 36, 39, 41, 46)	12	Staff
**0.94**	4.05	76	Number of Available Ventilators in each Hospital (41)	35	Equipment
**0.83**	4.12	76	Number of severe/moderate patients admitted in surgical departments in the last 24 hours in each hospital (8, 34)	73	Triage
**0.88**	4.10	76	Estimated Driving Time from Scene to Hospital (8-10, 29, 32, 36, 39, 47)	86	Transport

SD: standard deviation; * System and structures.

LOC: Level of Consensus; SD = Standard Deviation.

Considering the increasing complication and lack of certainty in many circumstances, assisting managers by providing quantitative models for them to facilitate decision-making and planning is critical (27). Providing quantitative criteria is a difficult task. Two studies have previously tried to identify the criteria influencing decision making in mass casualty incidents (8, 14).

The only study that has specifically addresses identification and prioritization of criteria affecting distribution of casualties following MCI is the study by Hall et al. (14) .This study used qualitative thematic analysis, identified 56 factor affecting patient distribution following MCIs and then prioritized the identified factor using modified Delphi method. One of the key features of this study is identification of experts who had peer-reviewed publications in the field of disaster management to participate in factor prioritization. However, some of the factors presented in this study can be separated to factors. For example, factors such as “Hospital characteristics” (ie, number, size, type, capacity, ownership, preparedness, experience), “Availability of transportation vehicles” (ie, ambulance, helicopter, bus, military, police, private vehicles with medical authorization, nonmedical vehicles)” and “Injury characteristics” (ie, number, type, severity)” may be separated to more definite factors and each definite factor weighted differently in Delphi rounds. 

 Some other factors, such as “Standard procedures for mass casualty incident” and “Teamwork and attitude”, are qualitative and general, and different conceptions of their meaning may exist. Considering the mentioned issues with the paper since its author suggested developing a decision support tool to assist first responders in casualty distribution following MCIs. In the present study, we eliminated the criterion (hospital capability) that were not objective by obtaining expert feedbacks and authors attempted to present and prioritize quantitative or objective criteria derived from previous systematic literature review. Therefore, it is believed that presented criteria are suitable for development of decision support tool for casualty distribution following MCIs.

Another study conducted by Adini et al. (8), aimed to develop a “load index model” to aid in decision making in mass casualty incidents. In this study, authors did a comprehensive literature review, performed a structured interview and then used modified Delphi for producing the shortlist of criteria related to patient distribution following MCIs. Although this study achieved some valuable results, sufficient information regarding its methodology has not been reported. This is also evident in the Delphi part of the study. In this regard, authors didn’t mention some main points including the procedures of comprehensive literature review, number of criteria extracted from the review, the process of structured review, details of experts in Delphi panel and number of Delphi rounds. However, it should be mentioned that mixed methodology was used and presenting all these parts in one paper would be challenging.

Despite the possibility of adding new criteria to presented Delphi forms, no additional criteria were proposed by experts. However, since the presented criteria in this study were extracted from a systematic literature review (7), this could be due to the comprehensiveness of the extracted criteria in this study.

**Limitations**

The most vulnerable part of Delphi studies might be “expert selection”. No globally accepted criteria exist for the required number of experts that should be selected and their characteristics in Delphi studies. In this study, as explained earlier, researchers set some criteria for selecting experts. 

Another limitation of Delphi studies is the level of consensus. Considering the importance of the topic, researchers set a level of consensus. In this study, we used a set point that was recommended by most literature. In order to resolve this problem, all accepted criteria have been sorted and the level of agreement for each criterion, whether accepted or rejected, was displayed in the table.

Presenting a large number of statements (114) could be counted as a study limitation and as described earlier, it could be the main cause reduction in the number of participants in the second round; therefore, it was possible to complete the online questionnaire in several sessions.

## Conclusions:

Following MCIs, casualty distribution between a number of healthcare centers is challenging. Many factors could influence decisions in this regard. Comprehensive identification of effective criteria in this critical task can be very helpful. However, for accelerating decision making regarding casualty distribution or in case of developing an agile decision support tool, it is necessary to use criteria that have a higher effect. In this modified Delphi study, the criteria that have been identified as influential on the distribution of casualties following trauma-related MCIs, were prioritized. Since none of the criteria presented in this study can be ignored in casualty distribution, authors sorted all accepted criteria according to level of agreement. This sorted list could be used as a catalogue for developing a decision support system or tool for casualty distribution following MCIs. 

## Data Availability

Authors of this study declare that all data of the current study, except for personal information of participants, are available and will be provided upon request.
